# A new strategy for repeated application of adenovirus based vectors: proof-of-concept in rhesus macaques challenged with SIVmac239

**DOI:** 10.1186/1742-4690-9-S2-P335

**Published:** 2012-09-13

**Authors:** C Sun, L Feng, L Xiao, P Li, L Zhang, L Chen

**Affiliations:** 1 Guangzhou Institute and Biomedicine and Health (GIBH), Guangzhou, China; 2 Comprehensive AIDS Research Center, Tsinghua University, Beijing, China

## Background

It is well recognized that highly active antiretroviral therapy (HAART) can control HIV/AIDS and prolong patient’s life. However, HAART is associated with drug toxicity, drug resistant, and patient's affordability. Therefore, developing an effective therapeutic vaccine that could induce HIV-specific immune responses may provide a solution to reduce the need of antiretroviral therapy. Adenovirus based vaccines have been extensively evaluated as a vaccine vehicle for HIV/AIDS and a variety infectious diseases, however, it has been a major concern that anti-Ad5 neutralizing antibodies in general population or after the first dose of immunization can hinder the its practical application. The idea of using less prevalent adenovirus serotypes lack of long-term safety record including oncogeneicity. Moreover, any adenovirus serotype will inevitablely induce neutralizing antibodies after one single use which render the strategy of using more serotypes infeasible.

## Methods

In this study, we explored a novel “one-size-fits-all” strategy, namely AVIP (Adenoviral Vector Infected PBMC), to circumvent the attenuated efficacy of Ad-based vaccines due to anti-Ad immunity.

## Results

We demonstrated that this AVIP strategy can elicit SIV-specific responses in both Ad5-seropositive and Ad5-seronegative macaques. Interestingly, comparable SIV-specific but weaker Ad5-specific responses were elicited in Ad5-seronegative monkeys received AVIP immunization as compared to direct injection of Ad5-SIV vaccines. Then a cohort of Ad5-seropositive SIVmac239-infected monkeys received AVIP immunization with Ad5 vectors expressing SIVmac239 Gag, Pol and Env with HAART. After therapeutic immunization, SIV-specific response was induced in vaccinated monkeys whereas a slower responses were observed in non-vaccinated monkeys. The functional immunological profiles were also being detected, and we are also monitoring whether this immunological benefit would afford a virological benefit.

## Conclusion

Our goal is to further develop the AVIP strategy as a simple but practically effective method for repeated delivery of Ad5-based vaccines in humans for HIV and other diseases.

